# Crystal structure of Fis1 and Bap31 provides information on protein-protein interactions at mitochondria-associated ER membranes

**DOI:** 10.1038/s42003-025-08625-4

**Published:** 2025-08-06

**Authors:** Minh Duc Nguyen, Yonghyeok Kim, Seung-Hyun Bae, Soeun Kim, Hyun Ku Yeo, Nam-Chul Ha, Ginam Cho, Sunghyun Moon, Kwang-Hwi Cho, Hyonchol Jang, Seoung Min Bong, Byung Il Lee

**Affiliations:** 1 Research Institute, National Cancer Center, Goyang-si, Gyeonggi Republic of Korea; 2 Faculty of Pharmacy, Phenikaa University, Hanoi, Vietnam; 3 Department of Cancer Biomedical Science, National Cancer Center Graduate School of Cancer Science and Policy, Goyang-si, Gyeonggi Republic of Korea; 4 Department of Agricultural Biotechnology, Center for Food and Bioconvergence, Research Institute for Agriculture and Life Sciences, CALS, Seoul National University, Seoul, Republic of Korea; 5 Department of Pathology and Laboratory Medicine, Cedars-Sinai Medical Center, Los Angeles, CA USA; 6 School of Systems Biomedical Science, Soongsil University, Seoul, Republic of Korea; 7 Present Address: New Drug Discovery Center Daewoong Pharmaceutical, Yongin-si, Gyeonggi Republic of Korea

**Keywords:** X-ray crystallography, Structural biology

## Abstract

In eukaryotic cells, mitochondria and the endoplasmic reticulum (ER) form close contacts at mitochondria-associated ER membranes (MAMs), which are involved in diverse cellular processes. The outer mitochondrial membrane protein Fis1, known for its role in mitochondrial fission, has been reported to interact with the ER-resident protein Bap31. Here, we present crystal structures of the cytosolic domain of human Fis1 in two distinct conformations, along with a co-crystal structure of Fis1 bound to the C-terminal region of the Bap31_vDED domain. One Fis1 structure resembles monomeric yeast Fis1 and features a characteristic N-terminal “Fis1 arm” conformation, which may indicate an autoinhibitory function. In the co-complex, the Bap31_vDED region engages the convex surface of Fis1’s tetratricopeptide repeat (TPR) domain. These findings provide structural insight into the interaction between Fis1 and Bap31 at ER–mitochondria contact sites.

## Abstract (web summary)

Structural analysis of Fis1-Bap31 interaction reveals a weak but unique interface between 2 proteins, illuminating how MAM-associated proteins mediate organelle tethering and signaling at ER-mitochondria contact sites.

## Introduction

Mitochondria, known as the powerhouse of the cell, play important roles in a variety of functions regulating cellular physiology and signaling, such as energy production, lipid biogenesis, and triggering cell death signaling^1,2^. Mitochondrial morphology is highly dynamic and can be regulated by membrane contact sites with other organelles^3^.

In particular, the mitochondrial membrane and the endoplasmic reticulum (ER) form a specific type of microdomain termed mitochondria-associated membranes (MAMs)^4,5^. These two organelles continuously communicate at MAMs to modulate a wide range of cellular physiological and pathological processes^6^. Accordingly, MAMs are known to play important roles in the maintenance of cellular physiology, including ER stress, mitochondrial dynamics^7^, inflammation^8^, autophagy^9^, and apoptosis^10^. These functions result mainly from the exchange of metabolites such as calcium ions (Ca^2+^) and various phospholipids between these two organelles. The dysregulation of MAM functions contributes to the progression of various types of human diseases, including neurodegenerative disorders^11–13^, cancers^14^, immune responses^15^, and cardiovascular diseases^5^.

MAMs are formed by multiple MAM tethering protein complexes composed of outer mitochondrial membrane (OMM)-anchored proteins and ER membrane-anchored proteins, and the MAM gap is ~10−30 nm^16–19^. Several MAM tethering complex proteins are present at MAMs and participate in various functions beyond membrane tethering. For example, the inositol 1,4,5-triphosphate receptor (IP3R)−75 kDa glucose-regulated protein (GRP75)−voltage-dependent anion-selective channel protein 1 (VDAC1) complex has been shown to facilitate Ca^2+^ flux from the ER into mitochondria^20^. The mitofusin-1 (MFN1)−MFN2 complex participates in maintaining the structure of MAMs by forming heterodimers and contributes to the ER stress response as well as mitochondrial fusion^21,22^. The vesicle-associated membrane protein-associated protein B (VAPB)–protein tyrosine phosphatase-interacting protein 51 (PTPIP51) complex has been shown to promote lipid transfer and regulate Ca^2+^ homeostasis^23,24^.

Among the MAM tethering protein complexes, one complex composed of mitochondrial fission protein 1 (Fis1) and B-cell receptor-associated protein 31 (Bap31) has been shown to establish a cell death signaling pathway by releasing Ca^2+^ ions from the ER^25,26^. Fis1 is a single-pass integral membrane protein anchored in the OMM through its C-terminal transmembrane (TM) helix. In yeast, this process is related to mitochondrial fission by the formation of mitochondrial fission complexes with adaptor proteins (mitochondrial division protein 1 (Mdv1) or CCR4-associated factor 4 (Caf4)) and dynamin-related protein (Dnm1)^27^. Indeed, mitochondrial fission has been linked to apoptosis, suggesting that Fis1 plays a role in cell death^28^. Bap31, an alternative protein binding partner of Fis1, is an integral polytopic ER membrane protein. Its cytosolic tail is cleaved by caspase 8 (Casp8) to generate a proapoptotic fragment (p20Bap31)^29,30^. This cleaved form of Bap31 has been shown not only to interfere with Bap31-mediated protein trafficking at various levels but also to trigger rapid transmission of ER calcium signals to mitochondria via the IP3R−GRP75−VDAC1 system^29,31^. Interestingly, the Fis1−Bap31 complex acts as a platform that creates a bridge and recruits proCasp8, promoting Bap31 cleavage^26^. These reports have demonstrated that the physical association between Fis1 and Bap31 is strongly related to the transfer of an apoptotic signal. However, the detailed structure of the Fis1−Bap31 complex has not yet been revealed. Here, we report a crystal structure containing both the Fis1 cytosolic domain (Fis1_ΔTM) and a variant of the death effector domain of Bap31 (Bap31_vDED). We also determined the two crystal structures of Fis1_ΔTM with different characteristic structural conformations at each N-terminus. Our study provides a possible structural snapshot of a MAM tethering complex and of conformational changes in the Fis1 structure depending on the protein binding partners involved in mitochondrial fission or MAM tethering.

## Results

### Crystal structures of the human cytosolic domain of Fis1 reveal the Fis1 arm at the N-terminus

**Fig. 1:**
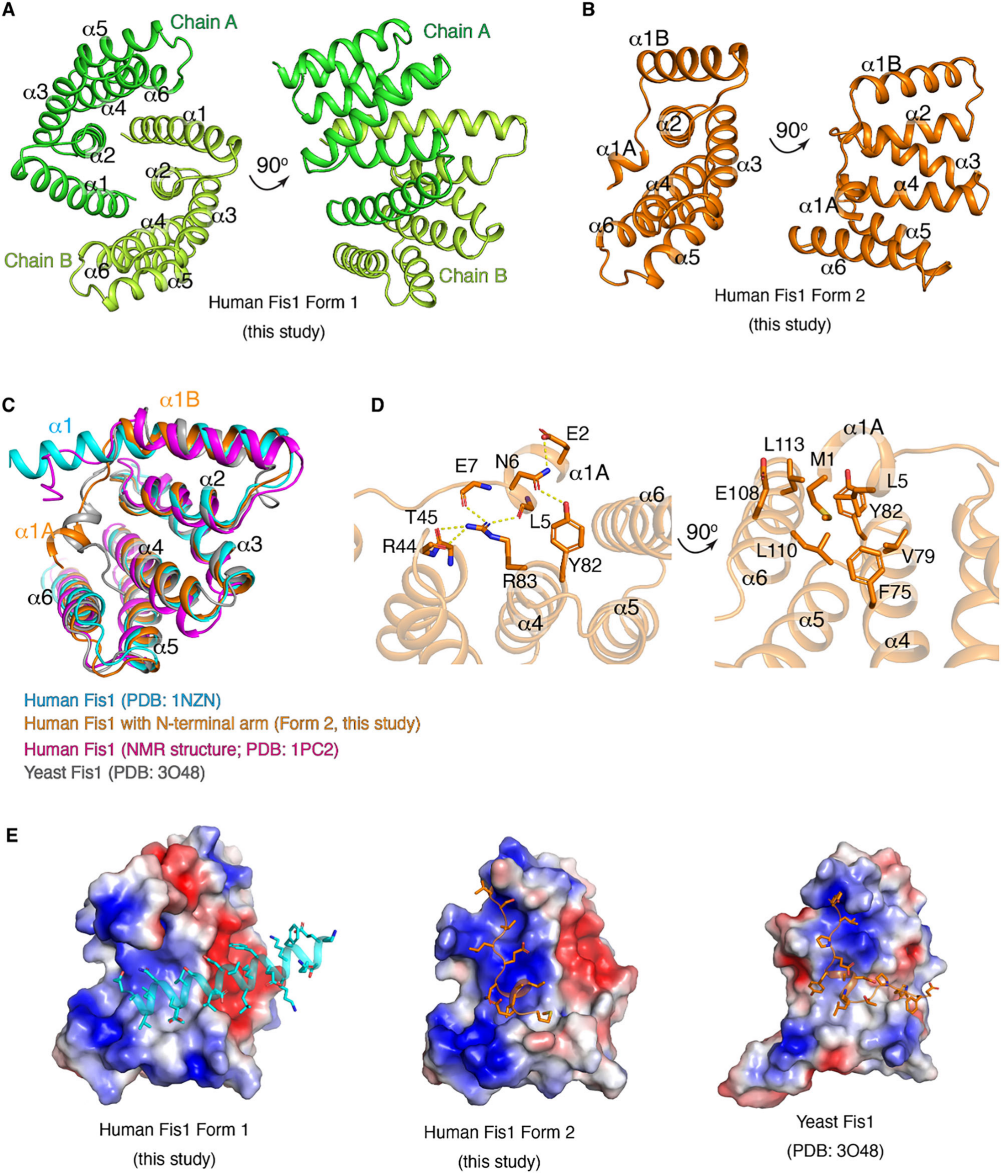
Crystal structures of the cytosolic domain of human Fis1 in different conformations. **A** New crystal structure of Fis1_ΔTM (Form 1) containing two Fis1_ΔTM molecules (green and light green) in the asymmetric unit with a structure identical to that of the previously reported Fis1_ΔTM structure (PDB code: 1NZN) except for the space group of crystal. **B** The new crystal structure of Fis1_ΔTM Form 2 reveals the presence of an N-terminal Fis1 arm. **C** Comparison of different structures of Fis1, human Form 2 (this study, orange), the previously reported crystal structure (cyan, PDB code: 1NZN), the previously reported NMR structure (partially covers the TM region, magenta, PDB code: 1PC2), and the previously reported yeast Fis1_ΔTM (gray, PDB code: 3O48). The α1 (cyan) of the previous structure, α1 A and α1B helices (orange) of the Form 2 structure were indicated. **D** The hydrogen bond networks that stabilize the N-terminal Fis1 arm (α1A) with α2 and α4 on the concave surface. The side chain and the main chains of the residues involved in the interaction are shown as sticks and lines, respectively. Hydrophobic interaction of Met1 and Leu3 in α1A with residues from the α4 and α6 helices. **E** The electrostatic surface potentials of the concave regions in the different crystal structures of Fis1_∆TM and human Fis1_∆TM Form 1 in this study. The long α1 helix of the neighboring Fis1_∆TM molecule in the dimer is shown in cyan (left). For human Fis1_∆TM Form 2 in this study (middle) and the yeast Fis1_∆TM (right, PDB code: 3O48) structures, N-terminal Fis1 arms from their own subunits are shown in orange.

We attempted to crystallize the Fis1−Bap31 complex with various constructs of purified proteins (Supplementary Fig. 1). Most of the diffracting crystals we obtained were identical to the previously reported crystal system of Fis1_ΔTM (P6_5_22 space group, containing one Fis1_ΔTM molecule in the asymmetric unit), which can form a putative Fis1 dimer through a crystallographic symmetry operation (PDB code: 1NZN)^32^. We obtained two additional forms of Fis1_ΔTM crystals belonging to different space groups (P2_1_ for Form 1 and P4_1_2_1_2 for Form 2; Table 1), which contained two or one Fis1_ΔTM molecule, respectively, in an asymmetric unit. The structure of the Form 1 crystal was dimeric Fis1_ΔTM, and the structure of the Form 2 crystal was monomeric Fis1_ΔTM (Fig. 1A, B).

**Table 1:** Data collection and refinement statistics

Crystals^a^	Fis1_∆TM(Crystal Form 1)	Fis1_∆TM(Crystal Form 2)	Fis1_∆TM+Bap31_vDED
Data collection			
X-ray source	PLS-5C	PLS-5C	PLS-5C
X-ray wavelength (Å)	1.000	1.000	1.000
Space group	P2_1_	P4_1_2_1_2	C222
Unit cell parameters (Å,°)	*a* = 44.82, *b* = 56.28 *c* = 57.01α = 90, *β* = 100.07, γ = 90.00	*a* = 56.07, *b* = 56.07, *c* = 97.66α = *β* = *γ* = 90.00	*a* = 104.63, *b* = 237.27, c = 90.62 *α* = *β* = *γ* = 90.00
Resolution range (Å)^b^	50.0‒2.30 (2.35‒2.30)	50.0‒1.70 (1.73‒1.70)	50.0‒2.69 (2.76‒2.69)
Total/unique reflections	74,451/12,419	147,301/17,973	259,435/31,733
Completeness (%)	99.4 (97.8)	99.9 (99.9)	99.8 (99.9)
I/σI	15.6 (2.4)	25.2 (3.6)	39.3 (1.16)
*R*_sym_ (%)^c^	8.5 (68.9)	7.4 (96.9)	4.7 (158.2)
Redundancy	6.0 (4.9)	8.2 (8.1)	8.2 (8.3)
Model Refinement			
*R*_work_/*R*_free_ (%)^d^	22.62/25.03	18.76/22.15	24.99/28.54
Number of nonhydrogen atoms			
Protein/Water	1971/10	1018/62	4732/25
Average B factor (Å^2^)			
Protein/Water	38.23/39.92	14.99/25.50	51.47/27.25
R.m.s. deviations from ideal geometry			
Bond lengths(Å)/Bond angles (°)	0.0012/0.31	0.018/1.57	0.007/0.91
Ramachandran plot (%)			
Favored/Allowed/Outliers	99.15/0.85/0	98.37/1.63/0	96.92/2.91/0.17
PDB code	7YKA	7YA9	8XWX

^a^Crystal contained Fis1_∆TM (residues 1−123) and Bap31_vDED domain (residues 168−233 + SAAA).

^b^Value in parentheses is for the highest-resolution shell.

^c^Rsym = Σh Σi | I(h)i – < I(h) > | / Σh Σi I(h)i, where I(h) is the intensity for reflection h, Σh is the sum for all reflections, and Σi is the sum for i measurements of reflection h.

^d^*R* = Σ| |Fobs| – |Fcalc| | / Σ |Fobs|, where Rfree is calculated for a randomly chosen 5% of reflections, which were not used for structure refinement, and Rwork is calculated for the remaining reflections.

Fis1_ΔTM exhibited the six antiparallel α-helical repeat bundle structure known as the tetratricopeptide repeat (TPR)-like fold for protein binding (Fig. 1)^32,33^. Overall, four helices (α2−α5) form two tandem helical TPR bundles (α2−α3 and α4−α5), generating a concave/convex surface with flanking capping helices (α1 and α6) (Fig. 1)^33^. The Form 2 structure of Fis1_ΔTM showed a similar fold to our dimeric Form 1 and to known human Fis1_ΔTM structures (with an RMSD of 0.597 Å for residues 1−123 aligned with 1NZN^32^, Supplementary Fig. 2A), except for the first 10 residues (residues 1−10) of the N-terminus (Fig. 1B, C). There was a large conformational change in the first 10 residues, corresponding to the N-terminal part of α1 helix (Fig. 1C and Supplementary Fig. 2A). This structural change was confirmed by the clear electron density map of the N-terminal region (Supplementary Fig. 2B). While the dimeric human Fis1_ΔTM formed a long α helix (α1) with approximately 32 residues (residues 1−32), approximately five residues in the middle of α1 (residues 7−11) were unfolded (Fig. 1B, C) in the monomeric Form 2 Fis1 structure, splitting into two helices, the α1A helix (residues 1−6) and the α1B helix (residues 11−27). The newly generated helix α1A bent toward the concave surface by ~42° (Fig. 1C) compared to the human Fis1 Form 1 structure with a long α1 helix reported under PDB code of 1NZN. This conformational change resulted in the formation of new interactions between α1A and α2, α4, and α6 of the same subunit (Fig. 1D). Interactions between N-terminal residues (Met1, Leu5, Asn6, and Glu7) and residues from the concave region (Arg44, Phe75, Val79, Tyr82, Arg83, Glu108, Leu110, and Leu113) stabilized the N-terminal structure (Fig. 1D). The concave surface of Fis1_ΔTM was positively charged and generated a suitable electrostatic environment to interact with the N-terminal end of α1 of the neighboring subunit or α1A of the same subunit (Fig. 1E). The solution structure of human Fis1_ΔTM (PDB code: 1PC2)^34^ was a monomer with a flexible N-terminal structure instead of a long α1 helix (Fig. 1C), and the crystal structures of yeast Fis1_ΔTM (PDB codes: 3O48, 2PQN, 2PQR, and 3UUX) exhibited a similar N-terminal conformation, termed the ‘Fis1 arm’, to our Fis1_ΔTM Form 2 structure^27,33,35^.

As described above, the long α1 helix of Fis1_ΔTM in the Form 1 structure is placed on a concave surface (formed by the α2, α4, and α6 helices) of the neighboring symmetric molecule, suggesting possible homodimeric associations (Fig. 1A, E)^32^. However, in our Fis1_ΔTM Form 2 structure, the concave surface of Fis1_ΔTM is occupied by its own N-terminal Fis1 arm instead of the long α1 helix of the neighboring Fis1_ΔTM molecule, inhibiting potential homodimer formation (Fig. 1B, E). The purified recombinant human Fis1_ΔTM exists as a monomer in solution, as determined by size exclusion chromatography (SEC) and AUC experiments (Supplementary Fig. 2C), although some crystal structures of human Fis1 (Fis1_ΔTM Form 1 structure in this study and PDB code: 1NZN) suggested possible dimerization. This AUC result supported that monomeric Fis1_ΔTM is in good agreement with previous biochemical studies and solution NMR structure studies of human and yeast Fis1 (this yeast Fis1 NMR structure includes a region of the TM)^32,34^. However, both chemical crosslinking and blue native polyacrylamide gel electrophoresis studies have shown that Fis1 can form homooligomeric complexes in mammalian cells^36,37^. Considering the two different Fis1 arm conformations in Fis1 structures, the N-terminal region of Fis1 may regulate oligomeric conversion by switching the interaction between the concave surface and its own Fis1 arm or the long α1 helix of another Fis1 molecule. Although crystal packing analysis of human Fis1_ΔTM Form 2 and yeast Fis1_ΔTM structures revealed additional possible dimer structures (Supplementary Fig. 3, left), considering the direction of the C-terminus containing the TM helix, the physiological relevance of these additional possible dimer structures with opposite TM directions is unclear. But one of the possibilities is that opposite TM orientation can stabilize the fission neck with two Fis1 molecules bridging across the neck during the mitochondrial fission.

### A crystal structure containing Fis1 and Bap31

Fis1 and Bap31 physically interact when they are overexpressed in mammalian cells, as shown by coimmunoprecipitation experiments^26^. However, our SEC experiments showed that Fis1_ΔTM did not coelute with various constructs of Bap31, probably due to its weak protein-protein interaction affinity (Supplementary Fig. 1B).

**Fig. 2:**
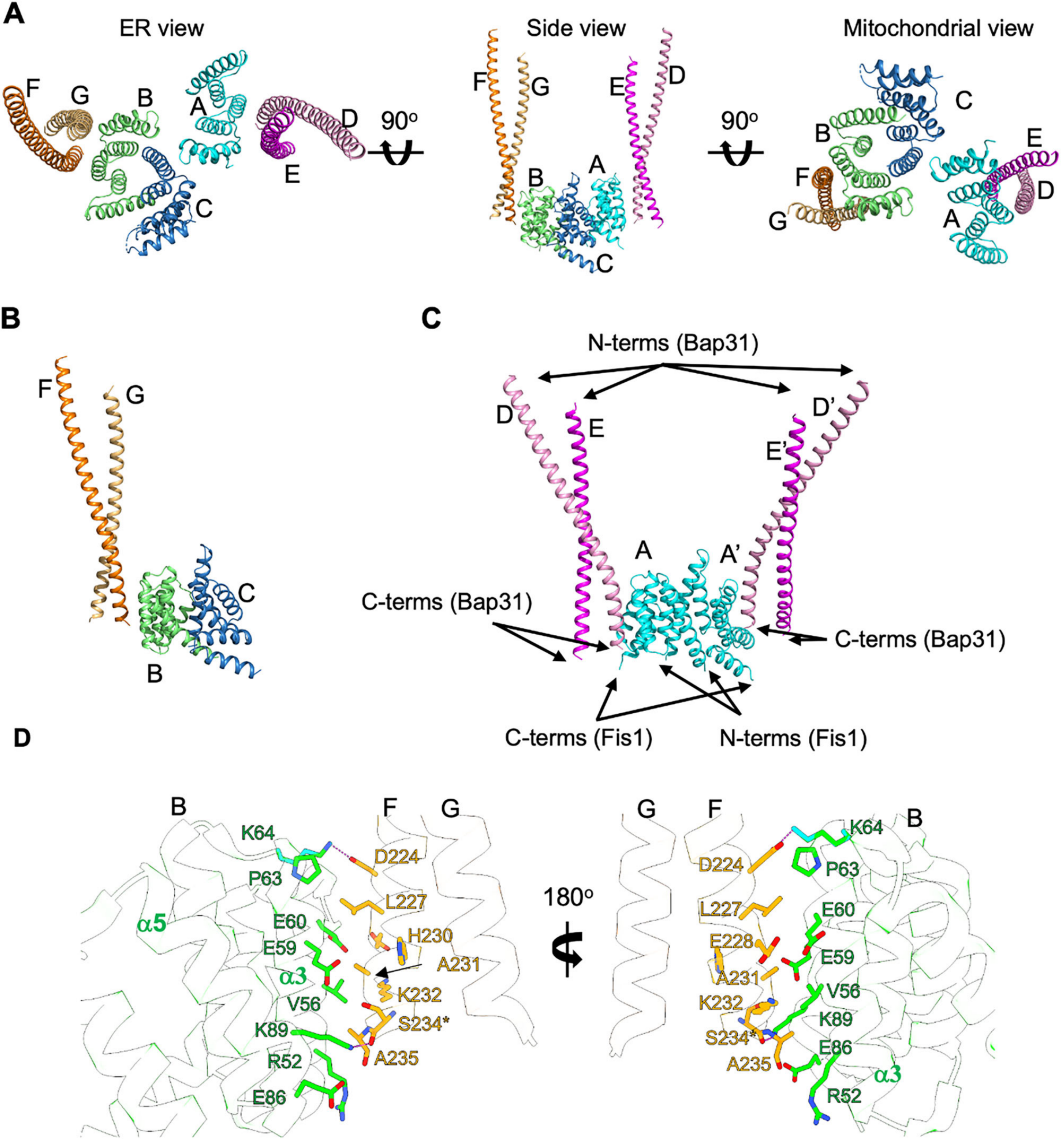
Crystal structure of the Fis1_ΔTM+Bap31_vDED. **A** The architecture of Fis1_ΔTM and Bap31_vDED in the asymmetric unit. Three Fis1_ΔTM molecules (chains A, B, and C are shown in cyan, green, and blue, respectively) and four Bap31_vDED (two dimers, chains D−E are shown in pink and magenta, respectively; chains F−G are shown in orange and light orange, respectively) in the asymmetric unit. **B** A representation of the Fis1_ΔTM dimer−Bap31_vDED CC dimer complex showing chain B−C of Fis1_ΔTM and chain F−G of Bap31_vDED. **C** Another representation of the Fis1_ΔTM dimer−Bap31_vDED dimer complex showing chain A−A’ (a symmetric mate of chain A), chain D−E, and chain D’−E’ (symmetric mates of chain D−E). **D** A detailed view of the contact residues between Fis1_ΔTM (chain B) and Bap31_vDED (chain F). *Ser234 is Gln234 in Bap31_WT, and a hydrogen bond is formed through the main chain of Ser234.

Although a stable recombinant protein complex was not available, we further tried to crystallize the Fis1−Bap31 complex by pooling all eluent fractions from SEC (Supplementary Fig. 1C) containing Fis1_ΔTM and various constructs of Bap31. The presence of both Fis1_ΔTM and Bap31_vDED in some crystals was confirmed by sodium dodecyl sulfate–polyacrylamide gel electrophoresis (SDS‒PAGE) (Supplementary Fig. 1D), and a crystal structure containing Fis1_ΔTM and Bap31_vDED (residues 168−233 + ^234^SAAA^237^; denoted as 168−233 + SAAA) was determined (Table 1). The crystal structure contained three molecules of Fis1_ΔTM (chains A, B, and C) and four molecules of Bap31_vDED (two Bap31_vDED dimers, chains D−E and F−G) in an asymmetric unit (Fig. 2A). It should be noted that we tried crystallization with a mixture of three proteins (Fis1_ΔTM, Bap31_vDEDs, and Casp8_tDED), but only Fis1_ΔTM and Bap31_vDED were found in the crystal, whereas Casp8_tDED was missing. Our effort to crystallize Fis1_ΔTM and Bap31_vDEDs without Casp8_tDED resulted in poorly diffracting crystals. We have no clear explanation for this result, and the role of Casp8_tDED in complex crystal formation is still unknown.

In the asymmetric unit of the crystal structure, two Fis1_ΔTM molecules (chains B and C, Fig. 2A, B) form a dimer with an extended α1 helix, as described above (Fig. 1A). Likewise, chain A of the Fis1_ΔTM molecule forms a dimer identical to that of its symmetrically related molecule (chain A’) (Fig. 2C). Two individual coiled-coil (CC) dimers formed by four Bap31_vDED molecules (chains D−E and F−G) interact independently with two Fis1_ΔTM molecules (chains A and B) in the asymmetric unit. Dimerization of Bap31_CC2 and Bap31_vDED was also confirmed by AUC experiments (Supplementary Fig. 4A). The remaining Fis1_ΔTM molecule (chain C) in the asymmetric unit did not interact with any Bap31_vDED molecule (Fig. 2A, B). Notably, only a single helix in the Bap31_vDED dimer makes direct contact with Fis1_ΔTM, and Bap31_vDED forms an antiparallel four-helical bundle in the crystal, as shown in the Bap29_vDED structures (Supplementary Fig. 4B, C)^38^. It is difficult to determine the biological assembly of the complex from the crystal packing, and we cannot absolutely rule out the possibility of crystallization artifacts. Henceforth, we refer to our structure containing Fis1_ΔTM and Bap31_vDED as the Fis1_ΔTM+Bap31_vDED structure.

As mentioned above, Fis1_ΔTM molecules in the Fis1_ΔTM + Bap31_vDED structure form dimers in which the extended α1 helix of Fis1_ΔTM packs against the concave surface of the neighboring Fis1_ΔTM molecule (Figs. 1 and 2A, B). The concave surface of Fis1 did not participate in the Fis1−Bap31 interaction. The C-terminal region (from Asp224 to the C-terminal end) of the Bap31_vDED helix in the CC dimer bound to the convex side of Fis1_ΔTM, which consists mainly of α3 and α5 helices (Fig. 2D). Since the buried surface area was only 273–361 Å^2^ (~3.4–5.3% and ~4.5% of the total surface areas of the Fis1_ΔTM and Bap31_vDED structures, respectively), the interaction between Fis1_ΔTM and BAP 31_vDED is likely very weak, explaining the impaired complex formation in the SEC experiment. In particular, there is only one salt bridge and only one hydrogen bond between Fis1_ΔTM and Bap31_vDED: between the side chain of Lys64 (chain B, Fis1_ΔTM) and the side chain Asp224 (chain F, Bap31_vDED) form a salt bridge, and the side chain of Lys89 (chain B, Fis1_ΔTM) and the main chain carbonyl group of Ser234 (chain F, Ser234 is Gln234 in the wild type (WT) Bap31_vDED) form a hydrogen bond (Fig. 2D and Supplementary Table 1); when analyzed with chain B (Fis1_ΔTM) and chain F (Bap31_vDED). Additionally, six residues of Fis1_ΔTM (Arg52, Ile55, Val56, Glu59, Glu60, and Glu86) and six residues of Bap31_vDED (Leu227, Glu228, Ala231, Lys 232, Ser234 (in WT, this residue is Gln234), and Ala235) form an interface for the Fis1_ΔTM and Bap31_vDED interaction (Fig. 2D). Since the Bap31_vDED construct for complex protein crystallization does not contain ten residues in the C-terminal end of CC2 (residues 237−246), we cannot rule out additional interactions between the missing C-terminal end of Bap31 and Fis1. Although the interaction residues for the two protein-protein interaction interfaces (chain A (Fis1)−chain D (Bap31) and chain B (Fis1)−chain F (Bap31)) were not identical (Supplementary Table 1), the Fis1_ΔTM + Bap31_vDED structure was sufficient to assign the residues involved in the interaction and revealed the importance of the C-terminal end region of Bap31 for complex formation.

### In vitro validation of crystal structure of the Fis1−Bap31 complex

**Fig. 3:**
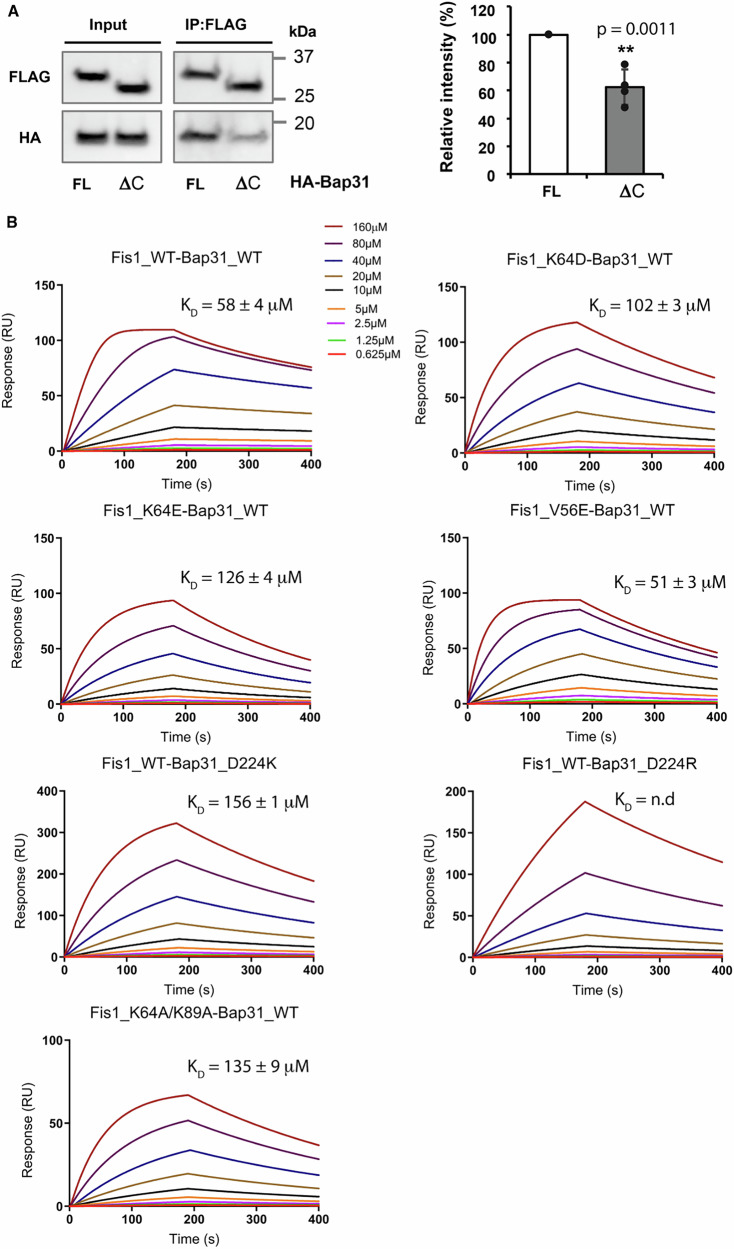
The interaction of Fis1−Bap31 in vitro. **A** The immunoprecipitation assay was performed with overexpressed full-length construct of FLAG-Fis1 and 2 different constructs of HA-Bap31 including full-length (FL) and C-terminal region truncated (ΔC), in the cells using FLAG-Fis1 as bait. Each dot in the graph (right) represents an individual biological replicate (*n* = 4), *p* value = 0.0011 (**); error bars represent standard deviation (SD). **B** The interaction of different constructs of Fis1_ΔTM and Bap31_CC2 was validated with SPR experiments by using purified recombinant proteins (WT and mutants of Fis1_ΔTM and Bap31_CC2). The SPR data were processed using equilibrium binding analysis instead of kinetic analysis due to the weak protein-protein interactions. All SPR experiments were performed in triplicate (*n*=3), and data are presented as mean ± standard error (SE). n.d: not determined. All source data are available in the Supplementary Information.

We aimed to examine the interaction between Fis1 and Bap31 by overexpressing their full-length forms in HEK293 cells. Using an immunoprecipitation assay with FLAG-Fis1 full-length as bait, we successfully detected the binding of HA-Bap31 full-length in the elution (Fig. 3A, left), confirming their interaction within the cells as previously observed^26^. Since the distal C-terminal domain of Bap31 (residues 224−233) is responsible for interacting with the convex surface of Fis1, based on Fis1_ΔTM+Bap31_vDED structure, we generated a truncated version of Bap31 with lacking this domain (ΔC). Co-immunoprecipitation assay was performed to evaluate the interaction between ΔC of Bap31 and Fis1 full-length. Although the interaction was not entirely eliminated, it was reduced by approximately 50% compared to the WT construct, highlighting the critical role of this domain in mediating the protein-protein interaction. (Fig. 3A and Supplementary Data 1).

Surface plasmon resonance (SPR) experiments were performed to further investigate the Fis1−Bap31 interaction using recombinant proteins lacking their transmembrane domains (Fig. 3B and Supplementary Data 1). While the CC1 domain of Bap31 (residues 124–167) failed to interact with Fis1 (Supplementary Fig. 5 and Supplementary Data 2), a weak but direct physical interaction was observed between Fis1_ΔTM WT (Fis1_WT) and Bap31_CC2 WT (Bap31_WT). The dissociation constant (K_D_) for the Fis1_WT−Bap31_WT pair was determined to be 58 (±4) μM, based on equilibrium analysis of SPR data.

To further explore the interaction, several key residues identified through structural analysis were mutated in both proteins. These included K64D, K64E, V56E, and K64A/K89A in Fis1, as well as D224K and D224R in Bap31. The mutations resulted in reduced binding affinities, with K_D_ values increasing approximately three-fold (102–156 μM) compared to the Fis1_WT−Bap31_WT pair, except for the V56E mutation, which had no significant effect (Fig. 3B). These findings underscore the importance of these residues in mediating the Fis1−Bap31 interaction.

Collectively, the SPR data support the structural insights from our crystal structure of the Fis1−Bap31 complex. They indicate that the convex side of Fis1 and the C-terminal region of Bap31 are critical for their interaction. Based on the in vitro validation, we believe that our Fis1_ΔTM+Bap31_vDED structure can be referred to as the Fis1_ΔTM−Bap31_vDED complex structure. Moreover, the complex structure provides a partial representation of the interaction as it occurs in solution.

### Conformational changes of Bap31_vDED upon interaction with Fis1

**Fig. 4:**
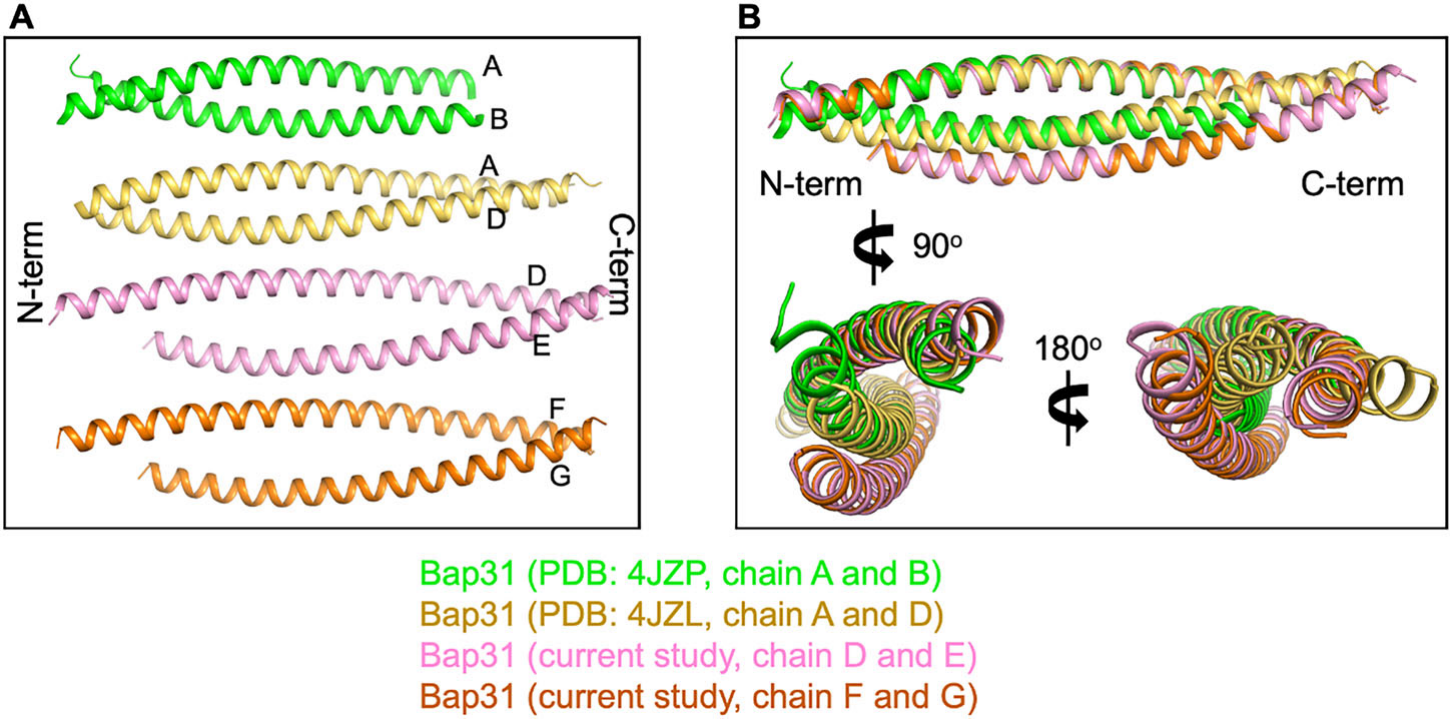
Conformational change in the Bap31_vDED domain upon complex formation with Fis1_ΔTM. **A** Overview of dimeric Bap31_vDED in a previous report and in the present study. Dimeric Bap31_vDED under acidic conditions (chains A and B, green, PDB code: 4JZP), dimeric Bap31_vDED under neutral conditions (chains A and D, yellow, PDB code: 4JZL), and two heterodimers of Bap31_vDED bound to Fis1_ΔTM (chains G−F and D−E, pink and orange, in this study) are shown. The terminal residues at the distal C-terminus of Bap31_vDED in the structural model are indicated in each chain. **B** Chain F of the complex structure superimposed on chain A of the previously reported Bap31_vDED structures, showing the displacement and movement of chain G relative to chain B and chain D.

Bap31_vDED has a dimeric CC structure^39^. This observation is consistent with our AUC data, which revealed that Bap31_vDED exists mainly as a dimer in solution (Supplementary Fig. 4A). It should be noted that while the C-terminal end region of Bap31 (residues 221−233) was not visible in the previously reported dimeric Bap31_vDED structure under acidic conditions (PDB code: 4JZP), three additional turns of helix (residues 221−233) can be defined and modeled in both our Fis1_ΔTM + Bap31_vDED structure and dimeric Bap31_vDED at neutral pH (PDB code: 4JZL) (Fig. 4). Interestingly, although Fis1_ΔTM + Bap31_vDED crystals were grown under acidic conditions (pH 4.5), the C-terminus of Bap31_vDED had a defined helical structure (Fig. 4A). These findings suggest that the C-terminal region of Bap31 is frequently disordered and that a helical structure is induced upon interaction with Fis1.

Upon superimposing the previous dimeric structures of Bap31_vDED (PDB code: 4JZP and 4JZL) with chain D or F of our Fis1_ΔTM + Bap31_vDED structure as a central figure, discernible displacements were observed in chains E and G in comparison to the second helix of Bap31_vDED CC in the previous structures (Fig. 4B). This suggests that the CC structure of Bap31_vDED remains stable and robust to differences in specific binding conditions.

### Comparison with mitochondrial fission complex structures from yeast

**Fig. 5:**
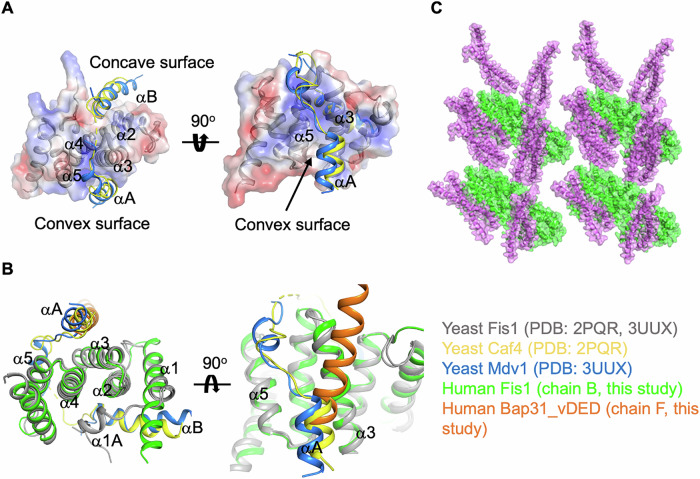
Comparison of the Fis1_ΔTM−Bap31_vDED complex structure in this study with a previously reported mitochondrial fission complex structure formed by Fis1 in budding yeast. **A** Overall structures of the yeast fission complexes Fis1−Caf4, Fis1−Mdv1, and Fis1 are shown as both electrostatic surfaces and cartoons to show concave and convex surfaces. **B** The Fis1_ΔTM (chain B, green)−Bap31_vDED (chain F, orange) structure is superimposed on the yeast Fis1 structure (gray) of the Fis1−Caf4 and Fis1−Mdv1 complex. Helices αA and αB of Caf4 (yellow, PDB code: 3O48) and helices αA and αB of Mdv1 (blue, PDB code: 3UUX) are presented. **C** The overall structures of Fis1_ΔTM (green) and Bap31_vDED (pink) in the crystal lattice mimicking the high-order oligomeric state of Fis1 in the mitochondrial membrane.

The crystal structures of the yeast mitochondrial fission complexes Fis1−Mdv1 and Fis1−Caf4 were compared with our Fis1_ΔTM−Bap31_vDED structure (Here, Mdv1 and Caf4 are adaptor proteins that recruit Dnm1 to Fis1). A similar binding mode was observed for the yeast Fis1−Caf4 and Fis1−Mdv1 complex structures, in which the αA helix of Caf4 or Mdv1 lies in a shallow groove lined by helices α3 and α5 (on the convex surface) of yeast Fis1 (Fig. 5A)^27,35^. The αA helix of the Caf4 or Mdv1 binding site overlaps well with the Bap31_vDED binding site of Fis1_ΔTM (Fig. 5B). In contrast to the αA helix, the αB helix of Caf4 or Mdv1 bound to the concave surface of yeast Fis1, and the Fis1 arm supported the αB interaction (Fig. 5A, B). These results suggested that a monomeric Fis1 molecule is wrapped by two adaptor protein α helices (αA and αB) to generate stable complexes with relatively high affinity when Fis1 functions in mitochondrial fission. At that time, both the convex and concave sides of the Fis1 TPR structure participate in protein-protein interactions (Fig. 5A)^27,35^. These structural differences suggest that the mode of protein-protein interaction for MAM association is quite different from that of protein complex formation for mitochondrial fission events.

Since the Fis1−Bap31 interaction was shown to be very weak, dimeric or even high-order oligomerization of Fis1, resulting in a locally concentrated protein-protein interaction patch, may be important for efficient mitochondria−ER membrane tethering with low protein-protein interaction affinity (Fig. 5C). The weak protein-protein interaction affinity of the MAM tethering protein complex has also been reported for the PTPIP51−VAPB complex^23^. These weak interactions may contribute to the dynamics of MAM formation and deformation.

### Modeled structure of the full-length Fis1−Bap31 complex

**Fig. 6:**
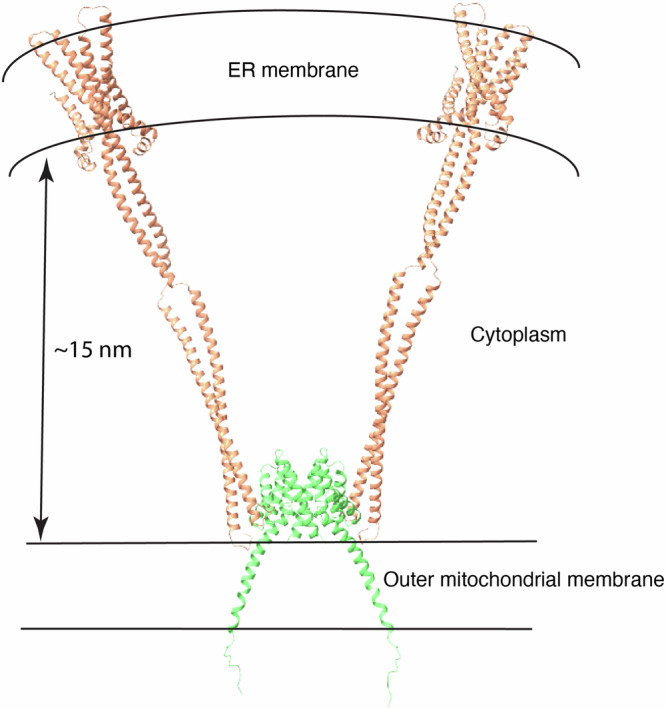
The modeled structure of the full-length Fis1−Bap31 complex. One dimeric Fis1 is shown in green, and two dimeric Bap31 proteins are shown in light orange.

We constructed a full-length Fis1–Bap31 complex model based on our structural data to illustrate the complete shape of Fis1–Bap31 complex. We generated full-length models of both dimeric Fis1 and dimeric Bap31. The Bap31 structure was predicted using AlphaFold3 (Supplementary Data 3) and subsequently refined through molecular dynamics (MD) simulations. As shown in Supplementary Fig. 6 (Left), we identified overlapping residues between the AlphaFold3-predicted model and the Bap31 crystal structure (PDB code: 4JZL), particularly at the terminal ends of helices. The central segment between the two coiled-coil domains, corresponding to residues DQLKKGAAVDGGKLDVGNA (residues 155–173), contains several glycine residues and exhibited moderate flexibility during the MD simulation, consistent with hinge-like motion. This flexibility may facilitate Bap31’s conformational adaptability at mitochondria-associated ER membranes (MAMs), where dynamic protein-protein interactions occur. The restrained regions remained stable throughout the 1 μs simulation, while the central region displayed subtle fluctuations. These minimal distortions were confined and did not propagate, validating the stability of the Bap31 dimer under near-physiological conditions. To assemble the complete complex, we combined one dimeric Fis1 molecule with two dimeric Bap31 molecules by superimposing our Fis1–Bap31 complex structure onto the full-length models (Fig. 6). According to the model, the distance for the cytosolic region in the complex model was ~15 nm, which is measured by the distance between two organelle membranes (Fig. 6) and is similar to the known gap distance of MAMs (10−30 nm). These findings suggest that the structural organization and flexibility observed in our model are compatible with the physiological dimensions and dynamic nature of MAMs.

## Discussion

Previous structural analyses of human (via NMR and X-ray)^32,34^, mouse (via NMR)^37,40^, and yeast Fis1 (via NMR and X-ray)^27,33,41^ have shown conformational differences in the N-terminal structure of Fis1. The Fis1 arm conformation is stabilized by intramolecular contacts with a concave surface formed by α2, α4, and α6 of Fis1. This structural feature aligns closely with the high-resolution crystal structure of Fis1_ΔTM Form 2 in our current study. In this structure, the first 9 residues at the N-terminus bend toward the concave surface and interact with its surface residues, as depicted in Fig. 1, stabilizing the ‘in’ conformation. These interactions are highly conserved across species, from yeast to mammals. This conservation is corroborated by previous structural and biochemical studies, providing additional support for the importance and universality of these stabilizing interactions^33,37,40,42^.

The deletion of the Fis1 arm reduces Drp1 (an ortholog of yeast Dnm1) recruitment to mitochondria, which impairs mitochondrial fragmentation and clumping, indicating the importance of the Fis1 arm in mitochondrial fission^40^. Fis1 and Drp1 are ubiquitously present in species with mitochondria. However, Mdv1/Caf4, which facilitate the formation of the mitochondrial fission complex, are fungus specific, and no known orthologs have been identified in vertebrates thus far. Notably, recent findings have demonstrated the involvement of Fis1 in Drp1-mediated fission events, particularly those that occur selectively at the periphery to eliminate damaged mitochondria^43^. Drp1 recruitment is known to be mediated by various mitochondrial membrane-anchored proteins, such as Mff, MiD49, and MiD51, during mitochondrial fission^43–46^. More recently, a direct interaction between Fis1 and Drp1 was also reported^42^. The Fis1 arm-dependent Drp1 recruitment suggests the importance of the Fis1 arm in the cellular functions of Fis1. The structures of human Fis1 identified in our study exhibit two distinct N-terminal conformations and suggest structure-dependent cellular roles: (1) preventing the dimerization of Fis1 and generating two protein binding sites at both the concave and convex surfaces for mitochondrial fission events and (2) generating multimeric Fis1 and creating a single protein-protein interaction site at the convex surface for MAM formation.

Intriguingly, the crystal structure of Fis1_ΔTM and Bap31_vDED in complex has been proposed to illuminate the protein‒protein tethering of MAMs. The complex structure shows an unexpectedly small protein interaction surface area between the distal C-terminal end region of Bap31_vDED and the convex region of Fis1_ΔTM. It remains possible that the interaction observed in the crystal structure represents just one of multiple possible binding modes, suggesting the Fis1−Bap31 interaction is dynamic and subject to regulation by other cellular components. Notably, only a single Bap31_vDED molecule in the CC2 of the Bap31 dimer was involved in the Fis1 interaction observed in the co-complex structure, indicating a very weak protein-protein interaction. Given that Bap31 functions at the ER and MAMs^47^, while Fis1 is primarily associated with mitochondrial fission, it is plausible that other scaffolding proteins play a role in stabilizing specific interaction states^25^. It is also conceivable that the interaction serves as a recruitment platform for other proteins, which in turn modulate the stability or function of the Fis1–Bap31 complex^26^. Taken together, additional regulatory proteins, post-translational modifications, or specific lipid environments at the MAM interface could further modulate the interaction in cellular context^48^.

Although we could not definitively confirm that our Fis1_ΔTM + Bap31_vDED structure is physiologically relevant, the immunoprecipitation, SPR results, and structural analysis suggest that the C-terminal region of Bap31 and the convex region of Fis1 are likely responsible for their interactions. Further studies employing mutagenesis, cryo-electron tomography, and in vivo functional assay could provide deeper insights into the structural and functional significance of these multiple interfaces in MAM organization. Investigating how additional MAM-associated factors influence Fis1–Bap31 interaction could also guide to clarify the role of this complex in organelle crosstalk and mitochondrial dynamics.

## Methods

### Cloning constructs

For structural and biophysical studies, all constructs were cloned and inserted into the pQlinkH vector using the BamHI and NotI sites (#13667, Addgene, USA)^49^, which provides an N-terminal polyhistidine tag followed by a TEV cleavage site. These constructs consist of the cytosolic domain of human Fis1, from which the C-terminal TM region is truncated (Fis1_ΔTM, residues 1−123); various domains of human Bap31, including the cytosolic domain with the TM deleted (Bap31_ΔTM, residues 124−246); coiled-coil 1 (Bap31_CC1, residues 124−167); coiled-coil 2 (Bap31_CC2, residues 168−246); four constructs containing variants of the death effector domain (Bap31_vDED, residues 168−234, 168−233 + SAAA [a tentative four amino acid sequence fused after Lys233], 168−236, and 168−238); and the tandem DED domain of Casp8 (Casp8_tDED, residues 1−188) (Supplementary Fig. 1A). We employed a single point mutation (Casp8_tDED_Y8A) in protein expression and purification to increase the solubility and stability of the recombinant Casp8_tDED protein^50^. Several polycistronic constructs, including Fis1_ΔTM−Bap31_CC2, Fis1_ΔTM−Bap31_vDED, Fis1_ΔTM−Casp8_tDED_Y8A−Bap31_vDED (four constructs as described above) and Fis1_ΔTM−Casp8_tDED_Y8A−Bap31_CC2, have been constructed for protein coexpression and copurification. All plasmid constructs were confirmed by DNA sequencing (Macrogen, Seoul, Korea).

### Protein expression and purification

Plasmids containing genes of interest were transformed into the *E. coli* strain Rosetta2(DE3) (Merck Millipore, USA) for protein expression. The cells were cultured in Terrific broth at 37 °C until the OD_600_ reached 0.6, and protein expression was induced by the addition of isopropyl-D-thiogalactoside (0.1 mM final concentration). After induction, the cells were cultured for an additional 16 h at 16 °C, harvested by centrifugation at 8000 rpm, and stored at −80 °C until use.

The cell pellet was resuspended in binding buffer (20 mM Tris-HCl [pH 8.0], 500 mM NaCl, 10% glycerol, 1 mM phenylmethylsulfonyl fluoride, 10 mM imidazole, and 4 mM β-mercaptoethanol) and lysed by a high-pressure homogenizer (Micronox, Sungnam, Korea). The crude lysate was separated by centrifugation at 13,000 × *g* for 2 h at 4 °C. The supernatant was incubated with 5 mL of Ni-NTA resin (Cytiva, Marlborough, MA, USA) for 30 min at 4 °C, followed by loading onto an open column. The resin was washed with washing buffer (20 mM Tris-HCl [pH 8.0], 200 mM NaCl, 5% glycerol, 20 mM imidazole, and 4 mM β-mercaptoethanol), and the protein was then eluted with elution buffer (20 mM Tris-HCl [pH 8.0], 100 mM NaCl, 300 mM imidazole, and 4 mM β-mercaptoethanol). Further clean-up was performed by size exclusion chromatography (SEC) using a HiLoad 16/600 Superdex 75 pg column (Cytiva, Marlborough, MA, USA) with SEC buffer (20 mM Tris-HCl [pH 8.0], 100 mM NaCl, and 0.5 mM tris(2-carboxyethyl) phosphine (TCEP)). For crystal screening, the polyhistidine tag was cleaved by incubation with the Tobacco Etch virus (TEV) protease at 4 °C overnight, followed by passage through Ni-NTA resin to capture the TEV protease and uncleaved proteins. The purities of the protein samples were verified by SDS‒PAGE.

### Protein crystallization

To elucidate the structure of the Fis1−Bap31 complex, we attempted to crystallize the protein complex by using various designed constructs (described in the Supplementary Information and Supplementary Fig. 1A). Despite extensive crystallization screening, we could not obtain protein complex crystals from mixtures of purified Fis1_ΔTM and Bap31_ΔTM or Fis1_ΔTM and Bap31_CC2. When we attempted crystallization with copurified protein mixtures containing Fis1_ΔTM, Casp8_tDED_Y8A, and four constructs of Bap31_vDED, protein crystals containing two different proteins were obtained (Supplementary Fig. 1A). Although we used three protein mixtures for crystallization, most of the crystals contained Fis1 only. Among them, crystals of dimeric Fis1_ΔTM (crystal Form 1 in Table 1) were observed in a reservoir solution containing 100 mM imidazole-HCl (pH 8.0), 200 mM zinc acetate, and 20% PEG3000, and crystals of Fis1_ΔTM with an N-terminal arm (crystal Form 2 in Table 1) were grown in a reservoir solution containing 100 mM CAPS-NaOH (pH 10.5) and 30% PEG400. Crystals containing both Fis1_ΔTM and Bap31_vDED were grown in a reservoir solution containing 100 mM sodium acetate (pH 4.6) and 1.5 M lithium sulfate (Supplementary Fig. 1). The Casp8_tDED_Y8A protein was not found in any of the protein crystals we obtained. Crystals containing Fis1_ΔTM and Bap31_vDED were observed after two weeks of crystallization. The crystals were cryoprotected by reservoir solution supplemented with 25% glycerol.

### X-ray diffraction and structure determination

X-ray diffraction data were collected at the 5C beamline at the Pohang Light Source (Pohang, Korea). Diffraction data were indexed, integrated, and scaled using HKL2000^51^. The Fis1_ΔTM crystals diffracted to 2.3 Å (Form 1) and 1.7 Å (Form 2) resolution. The molecular replacement (MR) phasing method was performed with the program PHASER in the PHENIX program package using the human Fis1_ΔTM (PDB code: 1NZN) structure as the initial search model^32,52,53^. Manual model building was performed using the program COOT^54^. Further refinement was performed by using Phenix. refine in PHENIX^55^.

Complex crystals obtained from protein samples containing longer constructs of Bap31_vDED (residues 168−236 or 168−238) possessed both Fis1_ΔTM and Bap31_vDED, but they diffracted poorly (only to ~8 Å). When Bap31_vDED was replaced with the shortest construct (residues 168−234), the crystals grown under the same conditions contained only Bap31 (100 mM sodium acetate (pH 4.6) and 1.5 M lithium sulfate). Therefore, we presumed that a small part of the Bap31 C-terminal region might be important for crystallization of the Fis1−Bap31 complex. Crystals with good diffraction were obtained when Bap31_vDED with a C-terminal amino acid tag (residues 168−233 + SAAA) was tested for crystallization. The resulting electron density map after MR search with the Fis1 structural model showed the presence of both Fis1_ΔTM and Bap31_vDED molecules in the asymmetric unit. Since the electron density map for Bap31_vDED was not good enough and the MR search failed with previous Bap31 structural models^39^, we manually generated a polyalanine model based on the initial electron density map for model building and then performed model refinement. Structural refinement was performed in phenix.refine^55^ in the PHENIX program suite. The details of the data collection and structure refinement are given in Table 1. The figures were prepared by using PyMOL Molecular Graphics System, Version 2.4.1 (Schrodinger) or ChimeraX ver. 1.6.1^56^. Many Bap31_vDED residues remained as alanine in the refined model due to the poor electron density map.

### Analytical ultracentrifugation

To determine the oligomeric state of Fis1_ΔTM, Bap31_CC2, or Bap31_vDED in solution, sedimentation velocity experiments were performed using a ProteomeLab XL-A analytical ultracentrifuge (AUC) (Beckman Coulter, Brea, CA, USA) equipped with UV/visible scanning optics. To perform the AUC measurements, the proteins were exchanged into storage buffer (20 mM Tris-HCl [pH 7.5], 150 mM NaCl, and 0.5 mM TCEP), and the protein samples were loaded into 12 mm double-sector cells with a quartz window and centrifuged at 42,000 rpm and an An-60Ti rotor at 20 °C for 500 min. Sedimentation velocity data were fitted to a continuous sedimentation coefficient (c(s)) distribution model using SEDFIT^57^.

### Surface plasmon resonance

Protein-protein interactions were also investigated by SPR (SR7500, Reichert, Buffalo, NY, USA). Two proteins, 200 μg of Fis1_ΔTM WT and 180 μg of Fis1_ΔTM mutants, were added to the chip surface in immobilization buffer (20 mM sodium acetate [pH 5.0]). Each protein was immobilized through standard amino acid coupling on a polyethylene glycol (PEG)-terminated alkanethiol (90%) and COOH-terminated alkanethiol (10%) sensor chip (Reichert, Buffalo, NY, USA) until saturation. SPR experiments were performed at 25 °C. The chips were equilibrated for 30 min with buffer (20 mM HEPES [pH 7.5], 100 mM NaCl, and 0.005% Tween 20) before SPR analysis. Various concentrations of Bap31_CC2 WT and mutants in buffer (20 mM HEPES [pH 7.5], 100 mM NaCl, and 0.005% Tween 20) were flowed over the Fis1_ΔTM WT- or mutants-immobilized chips at 30 μl/min for 3 min for association analyses. Subsequently, the running buffer was flowed over the chip for an additional 4 min (30 μl/min) for molecular dissociation analyses. Binding was monitored by the change in the refractive index at the surface of the chip, measured in response units (μRIU). A reference flow cell was used to record the response to bovine serum albumin (BSA) as a control, and the value of the response for BSA was subtracted from the value of the response for each sample. SPR data were fitted using Scrubber2 software with equilibrium analysis (BioNavis, Tampere, Finland).

### Cell culture and generation of overexpressing cells

HEK293FT cells (Thermo Fisher Scientific, Waltham, MA, USA) were cultured in DMEM (HyClone, Logan, UT, USA) supplemented with 10% heat-inactivated FBS, 1% penicillin–streptomycin, and 5 μg/ml Cellmaxin Plus (GenDEPOT, Baker, TX, USA) at 37 °C in a humidified atmosphere containing 5% CO_2_. Cells were co-transfected with FLAG-tagged Bap31 (WT full-length or ΔC mutant) and HA-tagged Fis1 (wild-type) using polyethylenimine (Polysciences Inc., Warrington, PA, USA). Two days post-transfection, the cells were harvested for immunoprecipitation assays.

### Immunoprecipitation and immunoblotting

Transfected cells were washed twice with cold PBS, lysed in RIPA buffer (Thermo Fisher Scientific, Waltham, MA, USA) containing a protease inhibitor cocktail (GenDEPOT, Baker, TX, USA), and mildly sonicated before centrifugation. The supernatants were subjected to immunoprecipitation using anti-FLAG M2 magnetic beads (cat. no. M8823, Sigma-Aldrich, St. Louis, MO, USA) following the manufacturer’s instructions, except that TRIS-buffered saline (50 mM Tris-HCl, 300 mM NaCl, pH 7.4) was used during the washing steps. Bound proteins were eluted with 3× FLAG peptide (Sigma-Aldrich, St. Louis, MO, USA). For western blotting, a rabbit anti-HA monoclonal antibody (1:1000; cat. no. 3724S, Cell Signaling Technology, Danvers, MA, USA) was used, followed by incubation with goat anti-rabbit IgG-HRP (1:5000; cat. no. 31460, Thermo Fisher Scientific, Waltham, MA, USA). For FLAG detection as a loading control, mouse anti-FLAG M2 antibody (1:1000; cat. no. F3165, Sigma-Aldrich, St. Louis, MO, USA) was used, followed by incubation with goat anti-mouse IgG-HRP (1:5000; cat. no. 31430, Thermo Fisher Scientific, Waltham, MA, USA). Immunoblot images were captured using the Fusion SL/SOLO imaging system (Vilber Lourmat, Marne-la-Vallée, France). Full immunoblots are shown in Supplementary Fig. 8.

### Molecular dynamics simulation

The full-length Bap31 homodimer structure was generated using AlphaFold3^58^ due to the lack of an experimentally determined structure. The full-length Bap31 dimer model was downloaded from the AlphaFold Server (https://alphafoldserver.com/) on May 13, 2024, using the model seed 1480019171 and included in Supplementary material. The predicted Bap31 model had ipTM, pTM, and ranking scores of 0.43, 0.45, and 0.65, respectively. A rectangular unit cell was constructed using CHARMM-GUI^59^, with the Bap31 dimer positioned at its center within 1-palmitoyl-2-oleoyl-sn-glycero-3-phosphocholine (POPC) bilayers ~120 × 120 Å in the x–y plane. The bilayer consisted of around 197 POPC molecules in the upper leaflet and 193 in the lower leaflet. The unit cell was filled with TIP3P^60^ water molecules along the *z*-axis, and 10 mM sodium (Na⁺) and chloride (Cl⁻) ions, along with their counterions, were added. The MD simulation was performed using NAMD^61^ (Version 3.b06) with the CHARMM36m^62^ force field. Prior to the production MD simulation, energy minimization and six equilibration steps were performed following the default settings in CHARMM-GUI’s “Membrane Builder”. During equilibration, constraint forces were gradually reduced, allowing lipid molecules to stabilize relative to their surrounding environment. The first two equilibration runs were executed in the NVT ensemble, followed by four runs in the NPT ensemble at 310 K and 1 atm, respectively. Temperature and pressure were maintained using Langevin thermostat and Langevin piston Nosé–Hoover^63,64^ methods. Electrostatic interactions were calculated with particle mesh Ewald (PME)^65^ approach, and van der Waals interactions were truncated using a switching function between 10 and 12 Å. Bond lengths involving hydrogen atoms were constrained via the P-LINCS^66^ algorithm, with a 2 fs integration time step. To stabilize structurally ambiguous regions, the predicted and crystal (PDB code: 4JZL) structures were aligned using Chimera (Version 1.17.3)^67^. Overlapping residues were identified at the terminal ends of two helices. Four consecutive residues (Glu152–Asp155 and Ala173–Lys176) from each chain were selected. Yielding a total of 16 residues subjected to dihedral angle restraints. The potential function applied for the dihedral angle restraints is defined as:

$${{\rm{U}}}({{\rm{x}}})={{\rm{k}}}(1+\cos ({{\rm{n \cdot x}}}-{{\rm{x}}} \_{{\rm{ref}}}))$$

where k is the force constant, set to 10 kcal/mol/rad².

After energy minimization and equilibration, a 1 μs production MD simulation was conducted under NPT conditions with periodic boundary conditions applied to maintain the integrity of the unit cell integrity.

### Generation of the full-length Fis1−Bap31 complex model

The full-length structural model of homodimeric Bap31 was generated using AlphaFold3^58^. The homodimeric full-length Fis1 model was created by adding a TM helix at the C-terminus of our Fis1 dimeric structure. Since the full-length Bap31 dimer exhibited a multidomain organization, its structural stability needed to be verified. To evaluate the structural stability of the AlphaFold3-predicted Bap31 model, we performed a 1 μs molecular dynamics (MD) simulation in a membrane and cytosolic environment. The simulation demonstrated that the overall fold of the model remained stable throughout the trajectory. The central segment−connecting the upper and lower helical groups−exhibited moderate flexibility, consistent with its proposed role as a hinge region. To prevent distortion of the terminal helices and maintain the overall structural integrity during the simulation, restraints were applied to selected residues. A detailed description of the restraint protocol and MD simulation is provided in Supplementary Fig. 6. The resulting full-length Fis1 and Bap31 structural models were then superimposed onto our Fis1–Bap31_vDED crystal structure.

### Statistics and reproducibility

Statistical analysis was performed using a Student’s *t*-test calculator (http://graphpad.com/quickcalcs/). The data are representative of at least three independent experiments for SPR, and at least four independent experiments for Western blot.

### Reporting summary

Further information on research design is available in the Nature Portfolio Reporting Summary linked to this article.

## Supplementary information

Description of Additional Supplementary Files

Supplementary information

Supplementary Data 1

Supplementary Data 2

Supplementary Data 3

Reporting summary

## Data Availability

Coordinates and structural factors were deposited in the RCSB Protein Data Bank with the following accession codes: 7YKA (crystal structure of Fis1_ΔTM Form 1), 7YA9 (crystal structure of Fis1_ΔTM Form 2), and 8XWX (crystal structure of Fis1_ΔTM + Bap31_vDED). The full-length dimeric Bap31 model predicted by AlphaFold3 is available in the Supplementary Information.
