# Supplementary material for: Crystal structure of Fis1 and Bap31 provides information on protein-protein interactions at mitochondria-associated ER membranes
Source: Commun Biol. 2025 Aug 6;8:1161. doi: 10.1038/s42003-025-08625-4 (PMC12328794; doi:10.1038/s42003-025-08625-4)
Supplement: Supplementary file 1 — Description of Additional Supplementary Files [file 42003_2025_8625_MOESM1_ESM.docx]

Description of Additional Supplementary Files

**File name:** Supplementary Data 1

**Description:** Data for Fig 3.

**File name:** Supplementary Data 2

**Description:** Data for Supplementary Fig 5.

**File name:** Supplementary Data 3

**Description:** AlphaFold 3 model for Full length Bap31.
