# Supplementary material for: Crystal structure of Fis1 and Bap31 provides information on protein-protein interactions at mitochondria-associated ER membranes
Source: Commun Biol. 2025 Aug 6;8:1161. doi: 10.1038/s42003-025-08625-4 (PMC12328794; doi:10.1038/s42003-025-08625-4)
Supplement: Supplementary file 2 — Supplementary information [file 42003_2025_8625_MOESM2_ESM.pdf]

Supplementary information

Crystal structure of Fis1 and Bap31 provides information on protein-protein interactions at mitochondria-associated ER membranes

Supplementary Figure 1

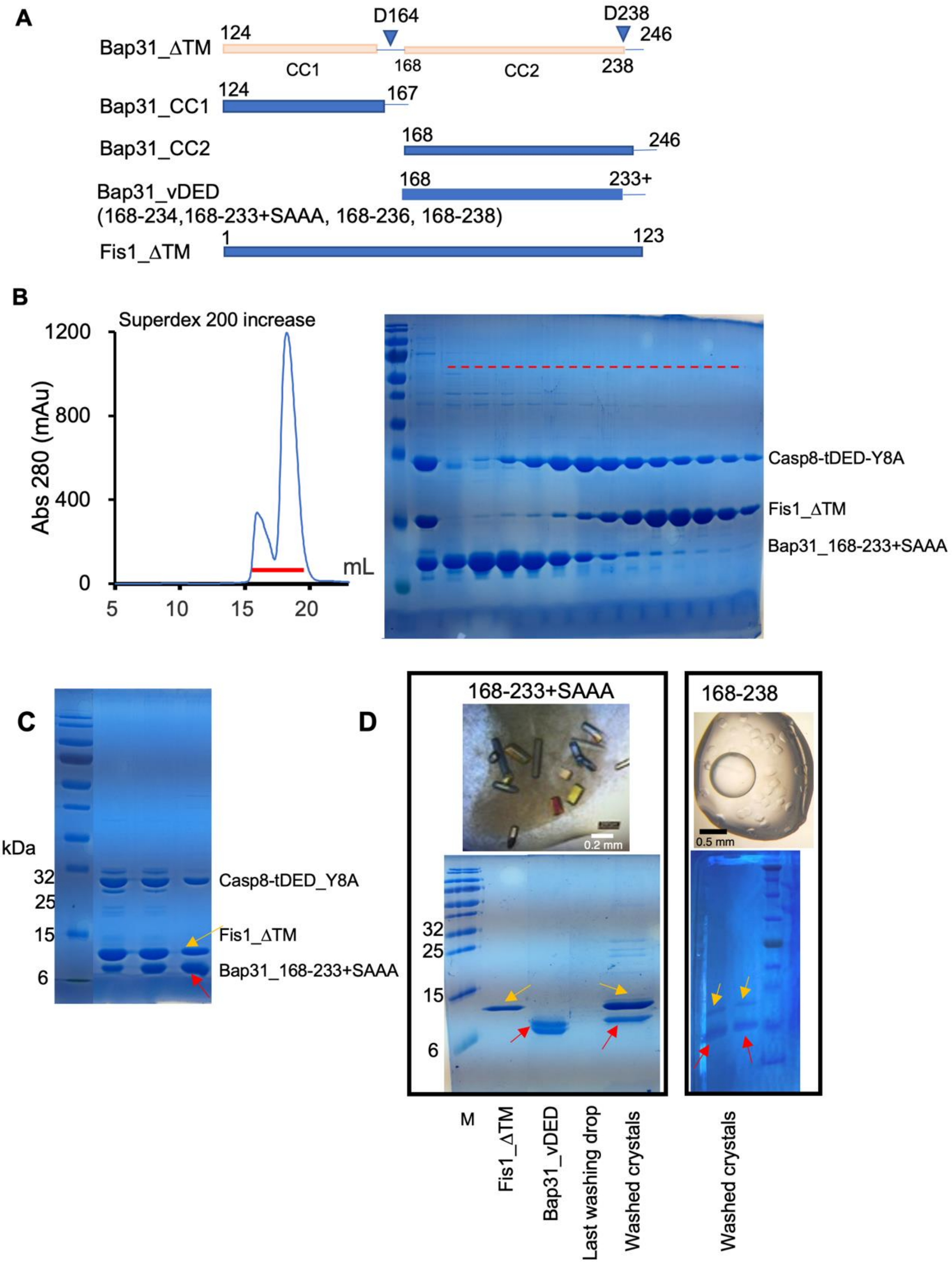

Supplementary Figure 1. Protein purification and crystallization. (A) Schematic diagram proteins used in this study. The two coiled-coil domains in Bap31_ΔTM are colored light orange, and the two caspase 8 cleavage sites (Asp164 and Asp238) are indicated by blue triangles. **(B)** Representative size exclusion chromatography and SDS–PAGE fraction analysis of coexpressed and copurified Fis1_ΔTM, Casp8_tDED_Y8A, and Bap31_vDEDs.

(C) Representative SDS–PAGE analysis of protein mixtures containing Fis1_ΔTM, Casp8_tDED_Y8A, and Bap31_168–233+SAAA constructs for crystallization screening. **(D)** Representative images and SDS-PAGE analysis of crystals from mixtures of Fis1_ΔTM, Casp8_tDED_Y8A, and Bap31_vDEDs (residues 168–233+SAAA [left panel], residues 168–238 [right panel]). The crystals were washed several times with reservoir solution before being dissolved in sampling buffer for SDS-PAGE analysis. The Fis1_ΔTM and Bap31_vDED bands are indicated by yellow and red arrows, respectively.

Supplementary Figure 2

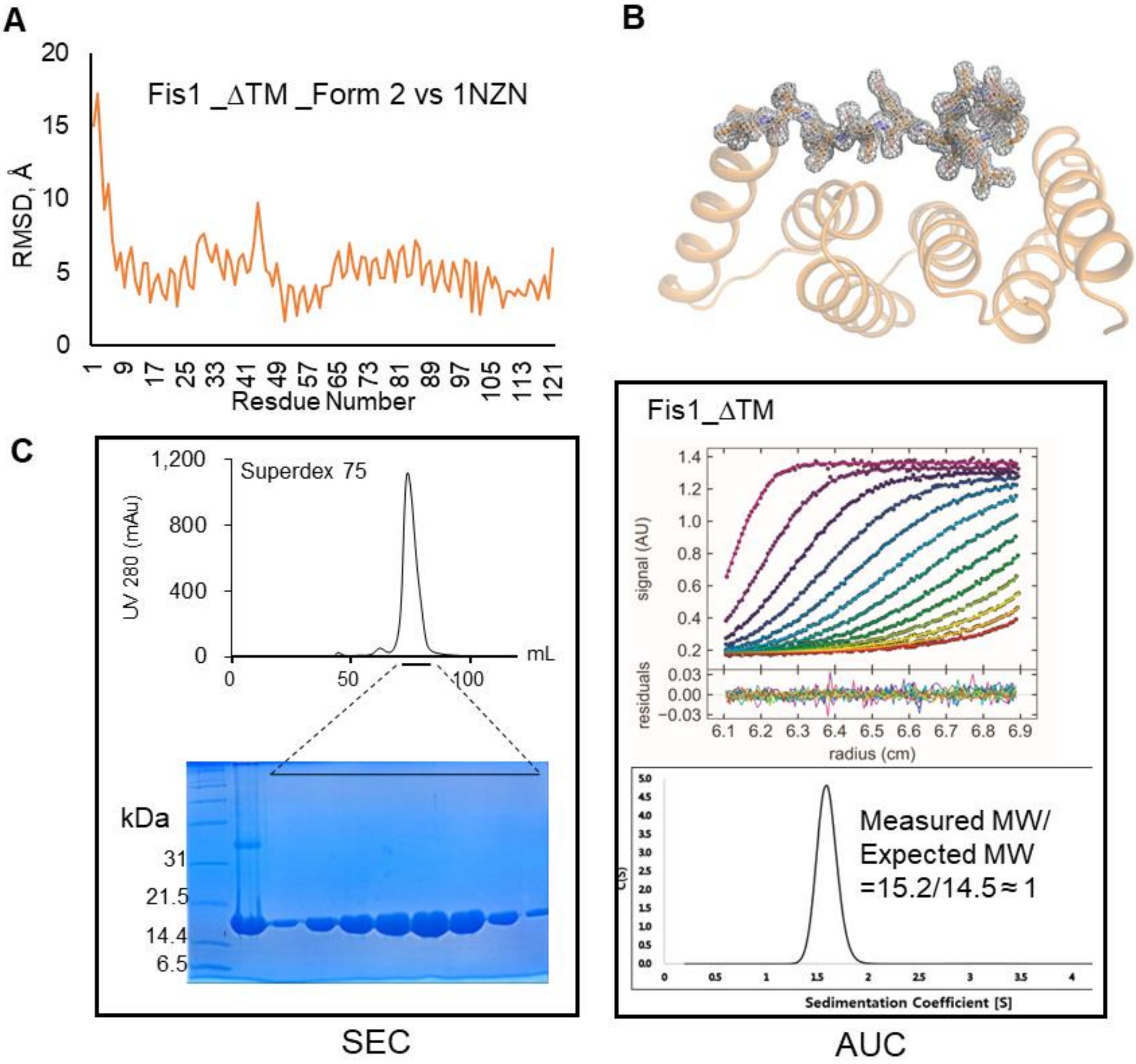

Supplementary Figure 2. Structural and biochemical features of the human Fis1 cytosolic domain. (A) RMSD comparison between Fis1_ΔTM Form 2 in this study and the previous crystal structure of human Fis1_ΔTM (PDB code: 1NZN). **(B)** The electron density map of the first 10 residues at the N-terminus of Fis1_ΔTM Form 2 in this study. **(C)** Oligomeric state of Fis1_ΔTM in solution. The gel filtration (Superdex 75 prep grade) chromatography results of Fis1_ΔTM (left) and AUC analysis (right) show that Fis1_ΔTM exists as a monomer in solution.

Supplementary Figure 3

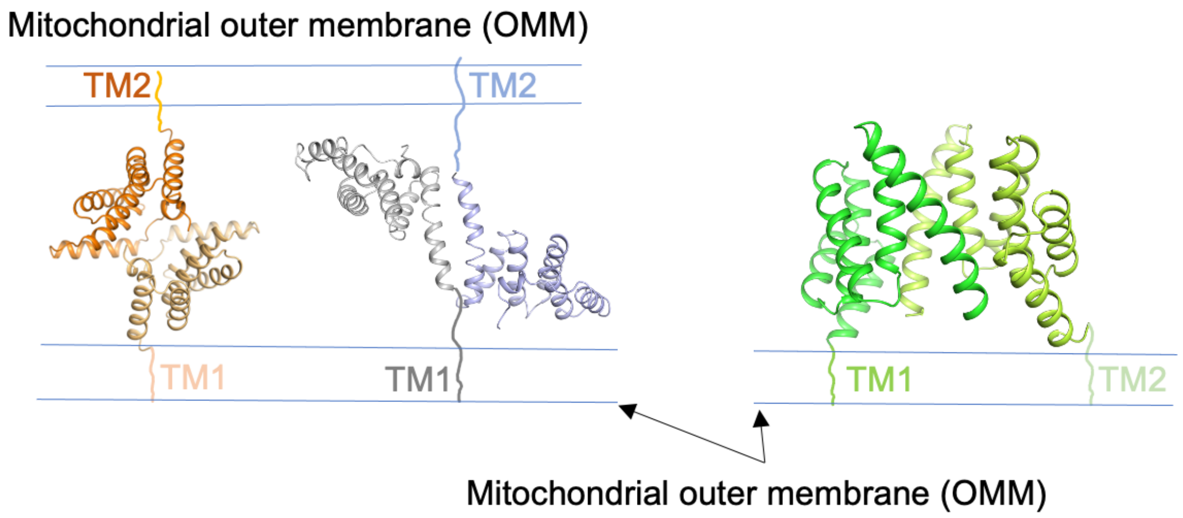

Supplementary Figure 3. Formation of different dimers of Fis1_ΔTM with the Fis1 arm. (Left) According to the crystal packing, in the presence of the N-terminal Fis1 arm, two Fis1_ΔTM molecules of human Fis1_ΔTM (light yellow and orange, Form 2, current study) or yeast Fis1_ΔTM (gray and light blue, PDB code: 3O48) can form loose dimers in which two integral C-terminal TM regions are extruded in an antiparallel direction. (Right) In the absence of the N-terminal arm, two Fis1_ΔTM molecules of human Fis1_ΔTM (green and light green; Form 1 in the present study and PDB code 1NZN) can form a compact dimer with parallel C-terminal TM helices.

Supplementary Figure 4

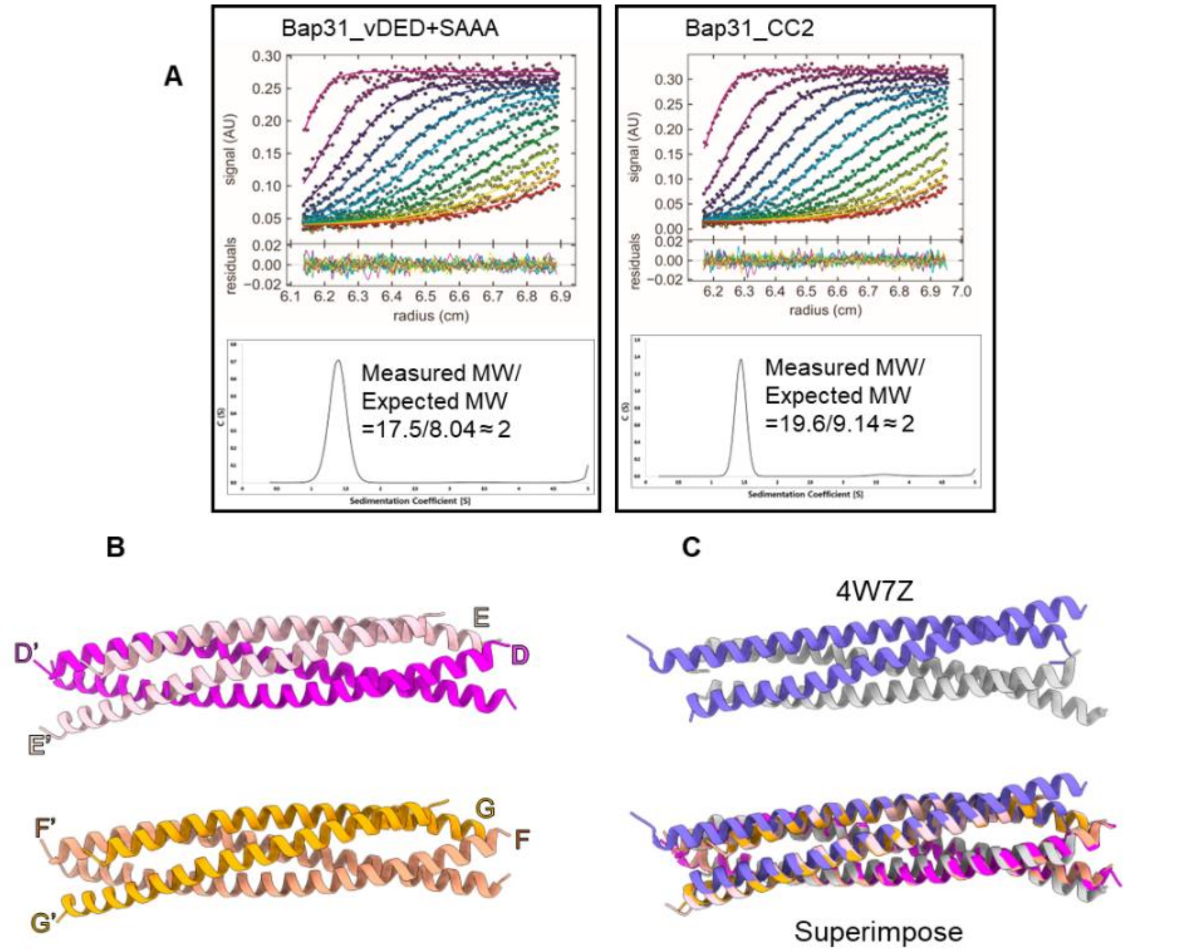

Supplementary Figure 4. Oligomeric state of Bap31 constructs. (A) AUC analysis of Bap31_vDED+SAAA (left) and Bap31_CC2 (right); both constructs suggested dimer formation in solution. **(B)** The dimeric Bap31_vDED forms an antiparallel tetramer with the same dimeric symmetric mate in the crystal structure of Fis1–Bap31_vDED; upper, D, D' (magenta)–E, E' (light pink); lower, F, F' (brown)–G, G' (orange) chains. The chain name is placed at the C-terminus of the corresponding chain in each panel. **(C)** (Upper) The crystal structure of Bap29_vDED shows the tetramer of two antiparallel Bap29_vDED dimers (medium blue and gray, PDB code: 4W7Z). (lower) The superimposition of these three tetramers shows a similar pattern in crystal packing.

Supplementary Figure 5

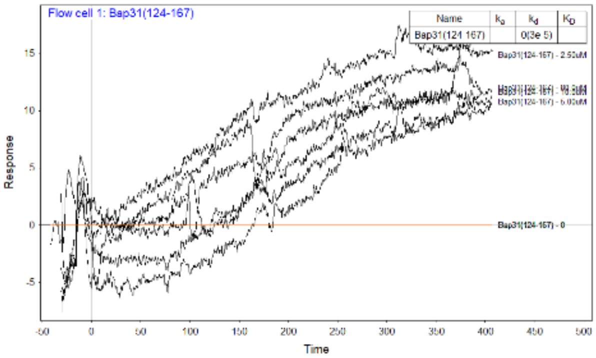

Supplementary Figure 5. Monitoring interaction between Bap31 CC1 and Fis1. An SPR experiment revealed no interaction between Bap31_CC1 and Fis1_ΔTM.

Supplementary Figure 6

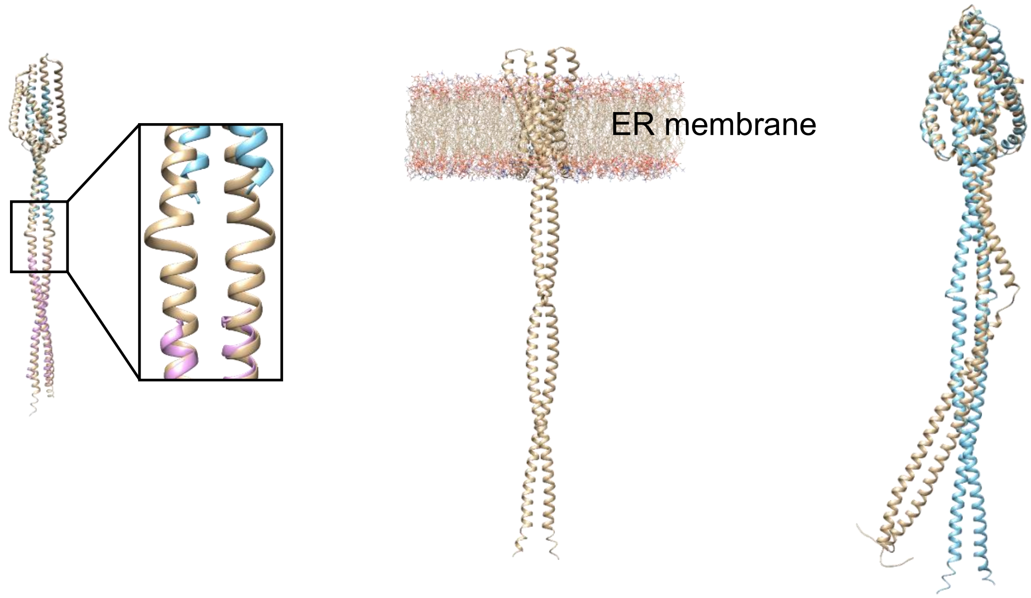

Supplementary Figure 6. Homodimeric full-length Bap31 model for modelling of full-length Fis1–Bap31 complex structure. (Left) Full-length Bap31 dimer structure generated by AlphaFold3 (brown) with the crystal structure of Bap31 coiled-coil (sky blue and light magenta; PDB code: 4JZL) superimposed. (Middle) MD simulation initial structure of the full-length BAP31 dimer in a membrane. (Right) Comparison of initial and final structures of the BAP31 dimer from the MD simulation. The sky-blue model represents the initial structure, while the light brown model shows the final structure after 1,000 ns of MD simulation.

Supplementary Figure 7

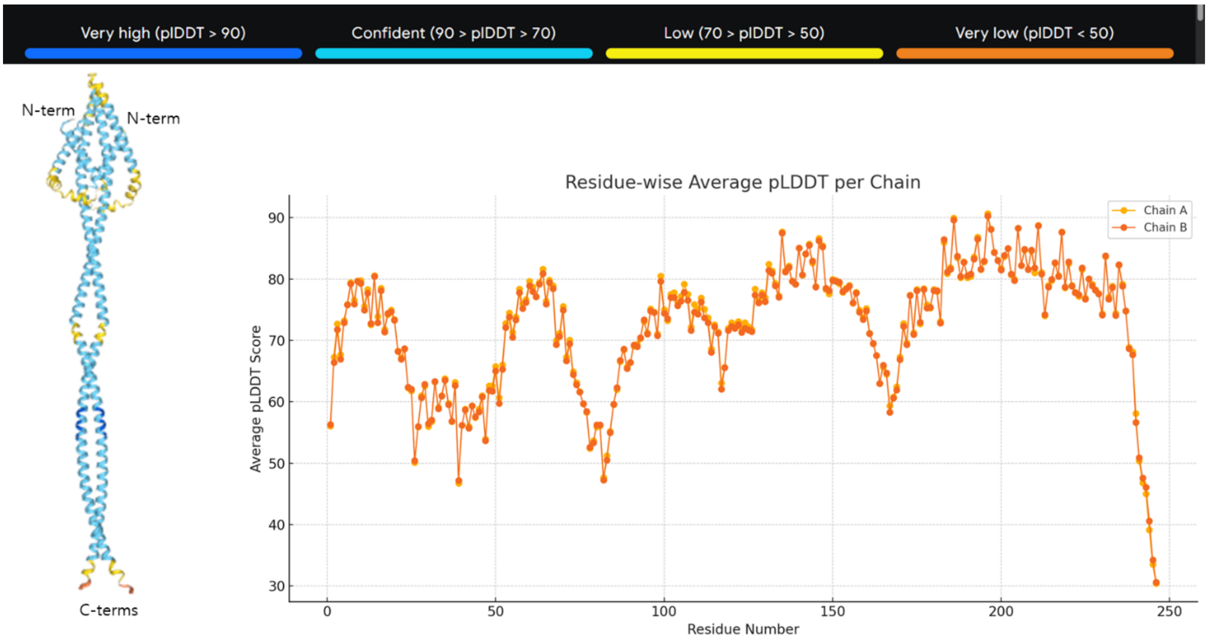

Supplementary Figure 7. AlphaFold3-based structural prediction and residue-wise confidence scores of full-length human BAP31 protein. (Left): Predicted 3D structure of Bap31 rendered by AlphaFold3. The color scale indicates the per-residue prediction confidence as pLDDT scores, with blue (pLDDT > 90) indicating very high confidence and orange (pLDDT < 50) indicating low confidence regions. (Right): Per-residue pLDDT scores across the full-length Bap31 sequence. The prediction confidence varies across domains, with large portions of the transmembrane helices and central domains showing moderate confidence (pLDDT 70–90), while terminal and loop regions show reduced confidence (pLDDT < 70).

Supplementary Figure 8.

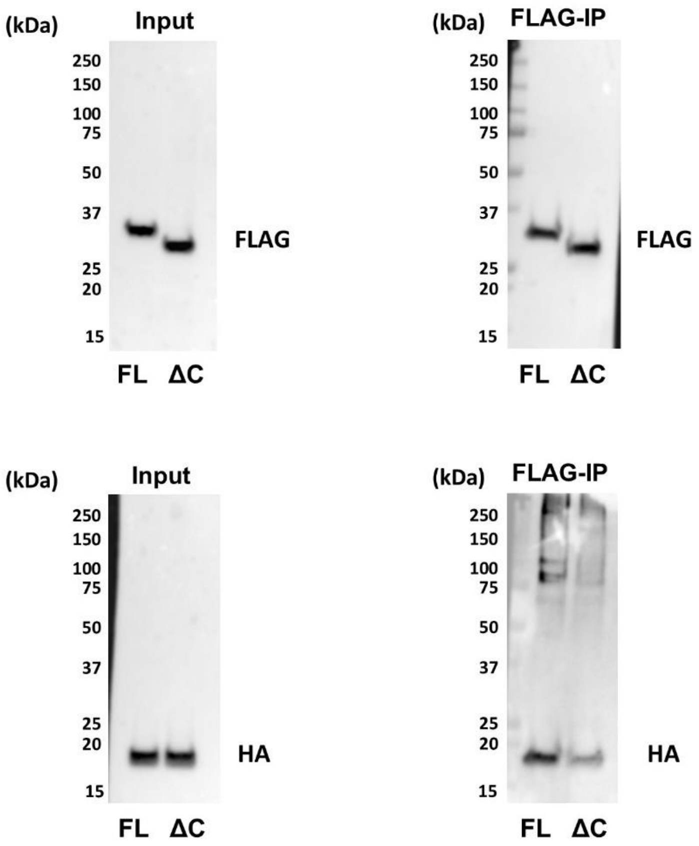

Supplementary Figure 8. Full immunoblots. Full-length images of western blot analyses in Figure 3.

Supplementary Table 1. Interface residues of Fis1–Bap31 interaction[a]

Fis1 (chain B)–Bap31 (chain F)			Fis1 (chain A)–Bap31 (chain D)		
FIS1	Interaction type[b]	Bap31	FIS1	Interaction type[b]	Bap31
Arg52		Ala235	Arg52		Ala235
Ile55		Ala235	Ile55		Ala231
Val56		Lys232	Val56		Ala231
Val56		Ala231	Val56		Glu228
Glu59		Ala231	Glu59		Leu227
Glu59		Ser234[c]	Glu60		Asp224
Glu60		Leu227	Glu60		Glu228
Glu60		Glu228	Glu86		Ser234[c]
Pro63		Leu227	Lys89		His230
Lys64	S	Asp224	Lys89		Ser234[c]
Lys89	H	Ser234[c]			

[a]Analyzed using the PISA (http://www.ebi.ac.uk/msdsrv/prot_int/cgi-bin/piserver) with PDB code of 8XWX for protein-protein or protein-ligand interactions and validate by visual inspection with Pymol or chimeraX, respectively.

[b]H, hydrogen bond; S, salt bridge; blank, interface residue with no other distinguishing characteristic.

[c]In WT, Ser234 is Gln234 in the WT and the interaction was through main chain of Ser234.
